# Study on the evolution and prevention of wellbore casing failure induced by marker bed water invasion

**DOI:** 10.1371/journal.pone.0351683

**Published:** 2026-08-21

**Authors:** Xianan Miao, Kangxing Dong, Jinbo Li, Delong Yu, Tingting Li

**Affiliations:** School of Mechanical Science and Engineering, Northeast Petroleum University, Daqing, Heilongjiang, China; Northeastern University, CHINA

## Abstract

Marker bed related casing failure under intensive water injection has become a major constraint during the late stage of compound flooding in oilfields, but the quantitative relationship among marker bed water invasion, formation weakening, and well-scale casing failure remains unclear. To address this issue, a typical well in the Sazhong development area of the Daqing Oilfield was investigated through theoretical analysis, laboratory experiments, and numerical simulation. Based on effective stress theory and a Drucker-Prager elastoplastic equivalent model, the strength degradation of the marker bed after water immersion was calibrated, and a numerical model for marker bed weakening and casing failure was established. The simulated minimum casing drift diameter differed from the field measurement by 7.2%, indicating that the model can reasonably reproduce the casing deformation observed in the representative well. The results show that marker bed strength decreases with soaking time, and abnormal water invasion is highly consistent with the intervals of casing failure. Failure is mainly concentrated at the interfaces between the marker bed and adjacent formations, where deformation incompatibility induces significant slip and subsequent casing deformation. Within the investigated parameter range, the sensitivity of the main controlling factors follows the order: injection pressure > marker bed thickness > marker bed dip angle > marker bed permeability. Injection pressure is the most direct controlling factor, whereas marker bed thickness is the dominant geological factor. Based on a minimum casing drift diameter threshold of 95 mm, zonal pressure control limits and corresponding operational recommendations are proposed for casing failure prevention.

## 1. Introduction

After more than 60 years of development, the Daqing Oilfield has entered the later stage of high water-cut production, during which wellbore casing failure has become an increasingly prominent engineering problem restricting efficient and stable oilfield production. The field has now entered its third peak period of casing failure, with more than 1,300 new casing failure wells reported annually [1–4]. With the continued implementation of water flooding, polymer flooding, and ternary compound flooding, formation seepage conditions, pressure field distribution, and the geomechanical environment have become increasingly complex. Under these conditions, casing failure induced by marker bed water invasion has become particularly prominent [1,2,4,5]. In typical blocks such as the Sazhong development area of Daqing, marker bed related casing failure wells account for 42.1% of all casing failure wells. Several concentrated casing failure zones have already formed, with a total area exceeding 38 km². In these zones, the casing failure rate reaches 90.1%, and most failures occur within 3–5 years. In addition, under conditions of increased salinity and fluctuating displacement pressure during compound flooding, the annual growth rate of casing failure is more than 30% higher than that during the water flooding stage [1,2,4]. This problem seriously disrupts injection production operations and restricts the effective development of geological reserves. Therefore, clarifying the evolution pattern and control mechanism of casing failure induced by marker bed water invasion under compound flooding conditions is of great theoretical significance and practical engineering value.

In early studies, shear casing failure associated with the marker bed was commonly attributed to the time dependent deformation and strength degradation of mudstone after water invasion [5]. However, field statistics show that creep alone cannot adequately explain the evolution of marker bed related casing failure, which is characterized by temporal concentration and local spatial clustering [1,2]. Subsequent studies have shown that the marker bed is commonly composed of oil shale or argillaceous shale rich in carbonates and bioclasts. Well developed bedding planes and weak interfaces make its tensile strength and fracture toughness strongly anisotropic [6,7], which provides the physical basis for the frequent occurrence of casing failure. On this basis, pore pressure diffusion and regional pressure variations after water invasion can further intensify shear action near the interfaces and aggravate local deformation incompatibility. This process amplifies the shear response of the casing [8–12] and eventually induces shear deformation and offset. Under fluid injection conditions, the evolution of fault-zone permeability, the nucleation of aseismic slip, and its unstable propagation can cause local stress redistribution and continuously intensify shear deformation, providing an important dynamic explanation for the evolution of marker bed related casing failure [13–16]. Field observations and numerical simulations generally indicate that these two failure modes dominate marker bed related casing failure [1,17–19]. Existing studies have provided an important basis for understanding marker bed related casing failure. However, most of them remain focused on statistical analysis, qualitative interpretation, or discussion of local mechanisms. A systematic quantitative description of the relationship among pore pressure evolution after marker bed water invasion, overall strength degradation of the rock mass, and the casing failure response at the well scale is still lacking [17–21]. This limitation is particularly evident under complex displacement conditions, where the effects and relative importance of engineering and geological factors on casing stress concentration, plastic accumulation, and drift diameter loss remain unclear [22–26]. As a result, existing prevention and control measures are not sufficiently supported in defining upper injection pressure limits or implementing zonal control.

Based on these gaps, this study selected a typical casing-failure well in the Sazhong development area of the Daqing Oilfield and established an integrated workflow combining field water-invasion monitoring, post-immersion weakening experiments, equivalent mechanical characterization, and well-scale numerical simulation. A Drucker Prager elastoplastic equivalent model based on effective stress theory was used to characterize the overall strength degradation, yielding behavior, and deformation response of the marker bed. On this basis, the effects of injection pressure, marker bed thickness, dip angle, and permeability on casing deformation were systematically analyzed, and the sensitive factors controlling the casing failure response were identified. Zonal pressure control limits and supporting operational recommendations were then proposed to provide a reference for the prevention and control of marker bed related casing failure under similar conditions.

## 2. Field data analysis and laboratory experiments

### 2.1 Field data analysis

**Fig 1:**
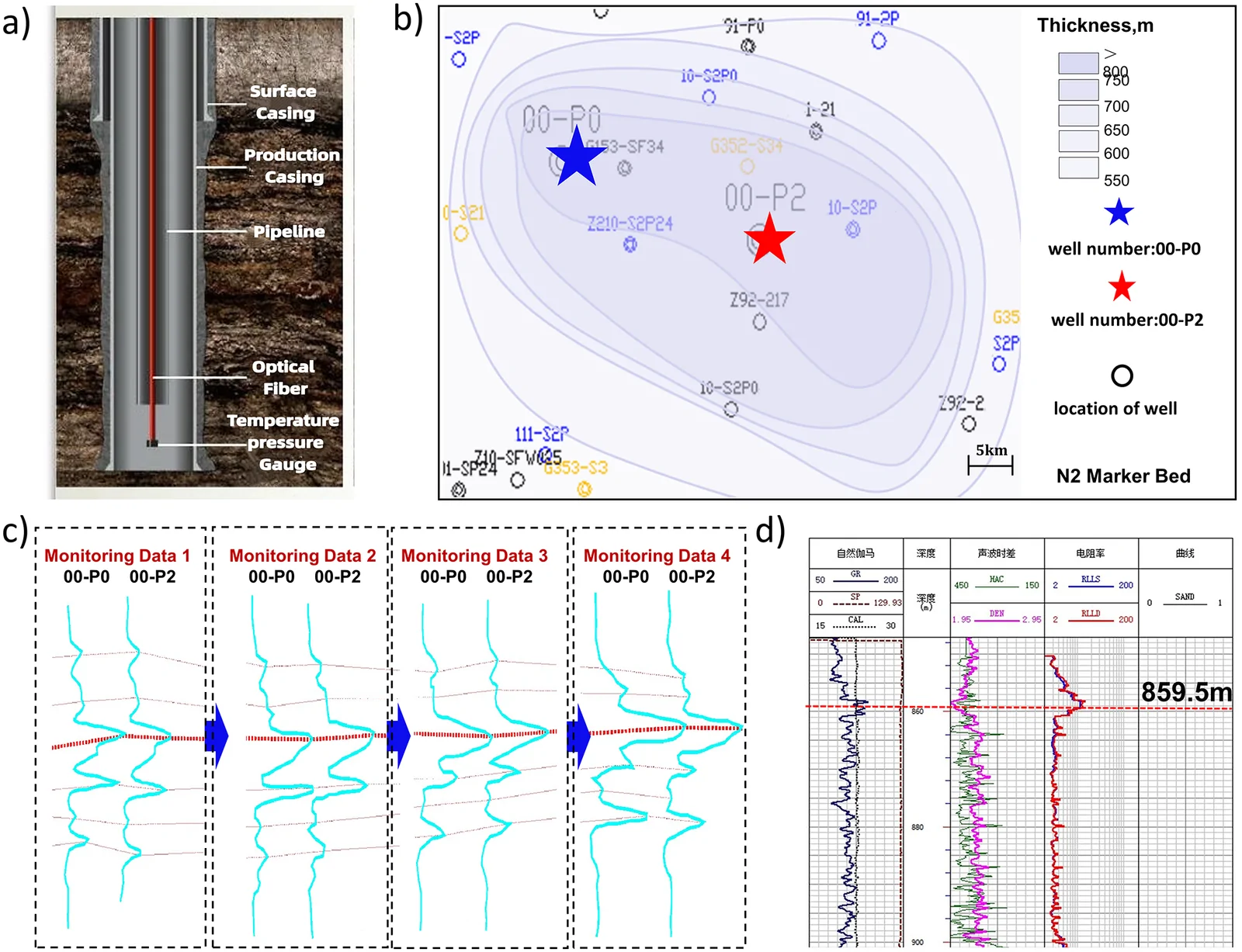
Field monitoring layout and response characteristics of marker bed water invasion. **(a)** downhole configuration of distributed fiber optic temperature monitoring; **(b)** spatial distribution of two casing failure wells and thickness distribution of the marker bed in the second member of the Nenjiang Formation; **(c)** changes in water invasion monitored four times over one year in two casing failure wells; and **(d)** logging curves of casing failure Well 00-P2.

Field monitoring was conducted in casing failure prone areas of the Daqing Oilfield. A distributed fiber optic temperature sensing system was deployed in the wellbore within the monitoring area to identify the occurrence and vertical distribution of water invasion within the formation. The downhole configuration of the distributed fiber optic temperature monitoring system is shown in Fig 1(a). Because injected fluid entering the formation causes local temperature anomalies, the temperature variation along the well depth can be used to identify the location and extent of water invasion in the marker bed and adjacent intervals. In the distributed fiber-optic temperature monitoring system, the entry of injected water into a permeable or weakened interval disturbs the original geothermal profile because the injected fluid has a different thermal state from the surrounding formation. Therefore, a persistent or repeatedly observed temperature deviation near the marker bed can be regarded as an indicator of preferential water invasion or enhanced water channeling in that interval.

Two typical wells were selected in the study area. Fig 1(b) shows the spatial locations of these two monitoring wells and the thickness distribution of the marker bed in the second member of the Nenjiang Formation. Both wells are located in areas where the marker bed in the second member of the Nenjiang Formation is well developed, indicating a strong correlation between marker bed thickness and the occurrence of casing failure. Fig 1(c) presents the quarterly monitoring results of water invasion over one year for the two monitoring wells. The abnormal water invasion near the marker bed can be seen to intensify gradually with time. Fig 1(d) shows the logging curves of Well 00-P2 at the time of casing failure. As shown in Fig 1(d), casing failure occurred in Well 00-P2 at a depth of about 859.5 m. Combined with Fig 1(c), it can be seen that the failure interval is highly consistent with the anomalous water invasion interval, indicating that abnormal water invasion in the marker bed is closely associated with the occurrence of casing failure.

Based on the above monitoring results, the marker bed in the lower part of the second member of the Nenjiang Formation is the main interval of casing failure development in the study area. Its water-invasion evolution shows a clear spatiotemporal association with the occurrence of casing failure, although the available monitoring data cannot fully resolve the strict causal sequence between water invasion and casing deformation. On this basis, Well 00-P2 was selected as the representative well for subsequent numerical model construction and validation. Its stratigraphic, logging, and casing failure data were then used to further analyze the evolution of casing failure induced by marker bed water invasion. The selection of Well 00-P2 was based on the clear spatial correspondence among the monitored temperature-anomaly interval, the well-developed marker bed in the second member of the Nenjiang Formation, and the later identified casing-failure depth.

### 2.2 Laboratory experiments and calibration of equivalent parameters

**Fig 2:**
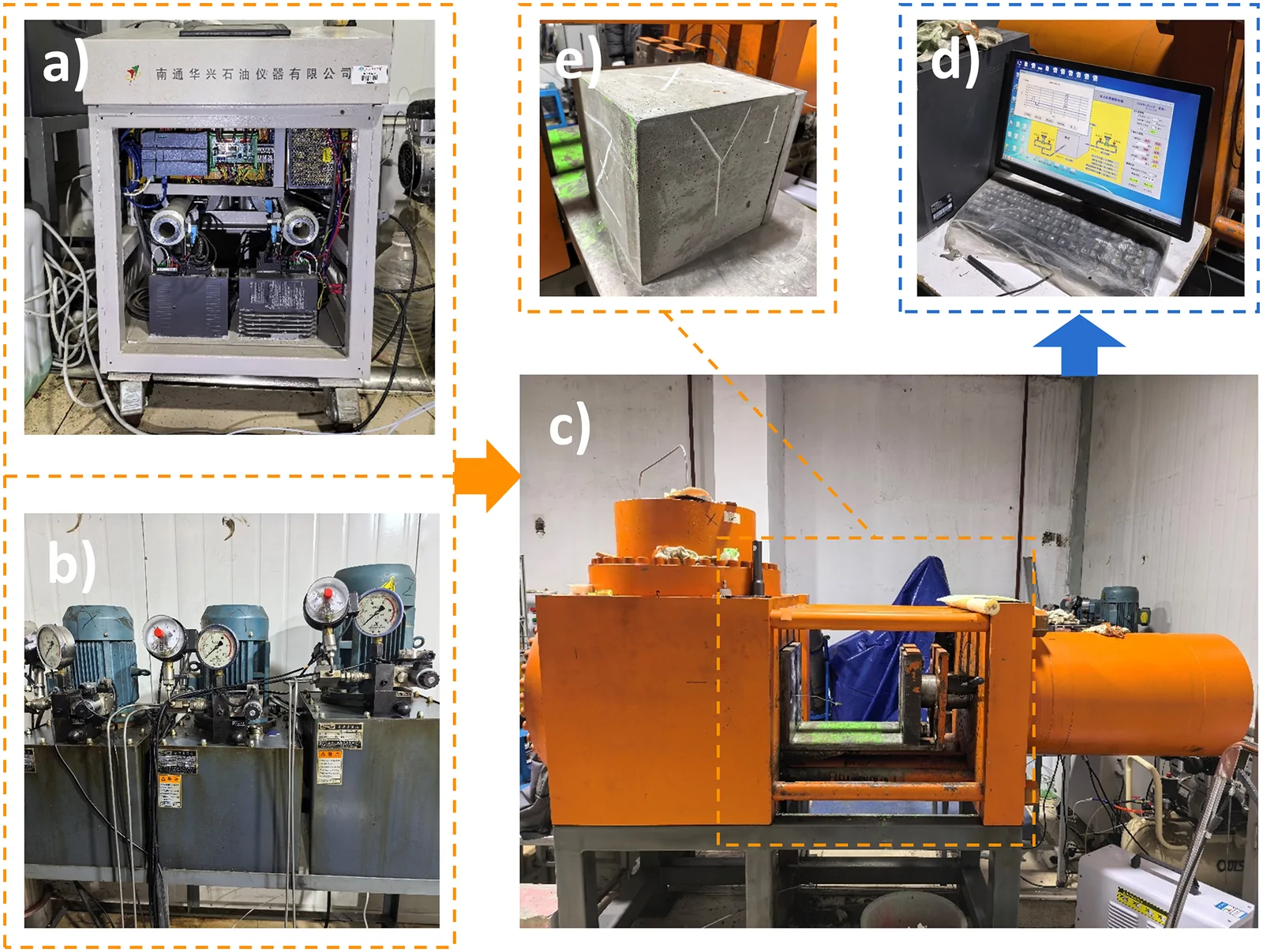
Experimental equipment. **(a)** constant pressure and constant flow injection system; **(b)** triaxial stress loading system; **(c)** true triaxial hydraulic fracturing apparatus; and **(d)** pressure data acquisition system.

**Fig 3:**
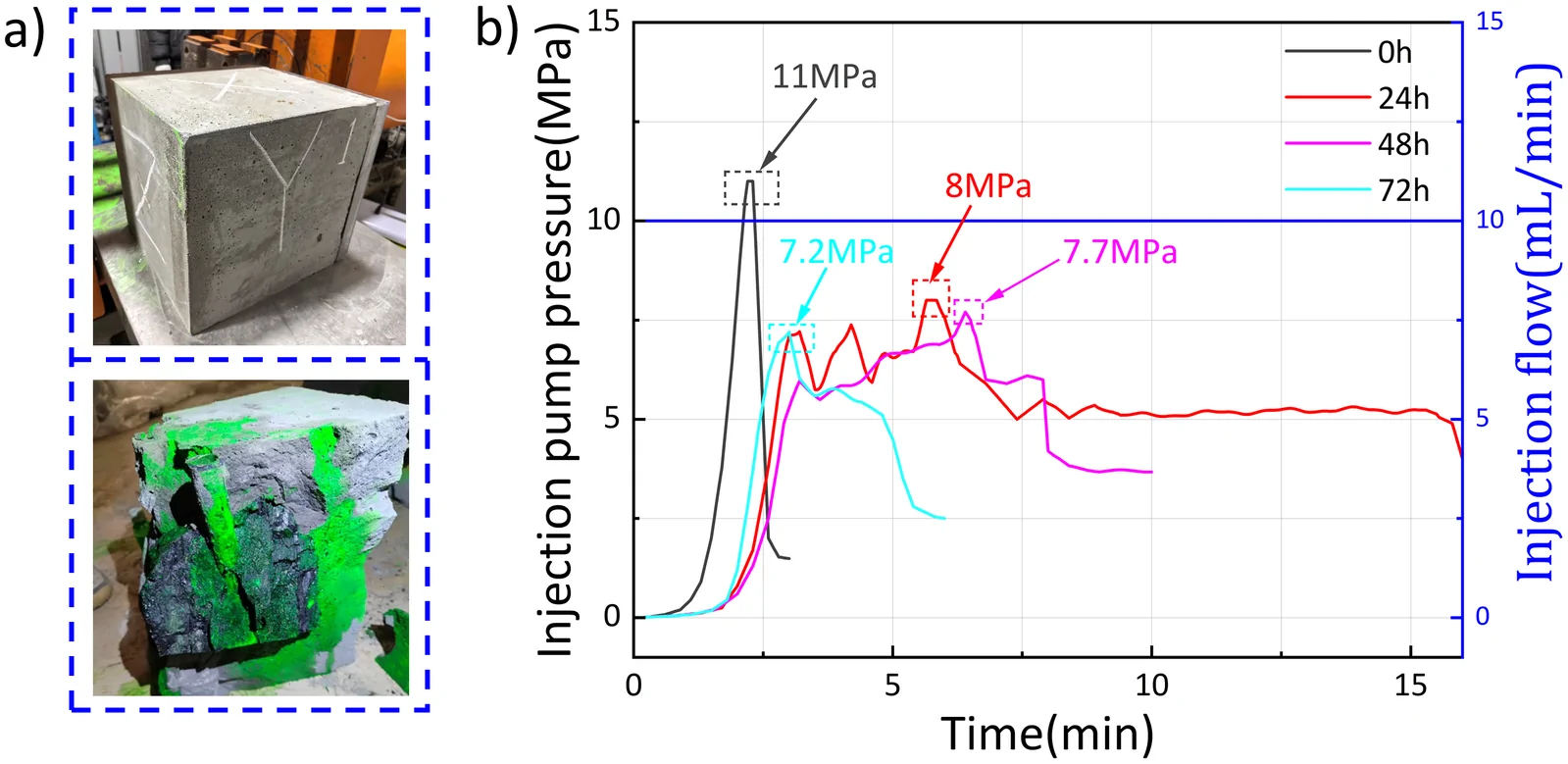
Experimental results. **(a)** specimens before and after hydraulic fracturing; and **(b)** real time fracturing pressure curves under different soaking conditions.

To investigate the strength degradation of marker bed rock under fluid soaking and to provide an experimental basis for the subsequent equivalent mechanical characterization of marker bed weakening caused by water invasion, cement wrapped hydraulic fracturing tests were conducted on marker bed cores. The weakening of the mechanical properties of the water invaded rock was quantitatively characterized by measuring changes in fracture initiation pressure. The experimental apparatus is shown in Fig 2. The test cores were taken from the marker bed in the lower part of the second member of the Nenjiang Formation at a depth of 850 m. Each core measured 150 mm × 150 mm × 150 mm, and cement was placed around the core to prepare specimens with dimensions of 300 mm × 300 mm × 300 mm. The cement layer was used to provide a stable external constraint and injection boundary, rather than to fully reproduce the in-situ wellbore environment. Four test conditions were designed. Condition 1 involved direct hydraulic fracturing, whereas Conditions 2–4 involved hydraulic fracturing after fluid soaking, with soaking times of 24, 48, and 72 h, respectively. Four specimens, as shown in Fig 3(a), were prepared for each condition, giving a total of 16 specimens, so as to reduce the influence of experimental scatter on the results.

After curing under standard conditions for 28 days, the specimens were subjected to hydraulic fracturing tests. The prepared specimens and the fractured specimens are shown in Fig 3(a). In the baseline test, hydraulic fracturing was conducted directly. The water weakening test was carried out in two stages. First, fluid was injected into the specimen. When the pressure reached 5 MPa, the injection was stopped, and the specimen was allowed to soak under natural pressure holding conditions for 24, 48, and 72 h, respectively. Hydraulic fracturing was then performed again. The measured fracturing curves are shown in Fig 3(b). Under the unsoaked condition, the fracture initiation pressure of the core was 11.0 MPa. After soaking for 24, 48, and 72 h, the corresponding fracture initiation pressures decreased to 8.0, 7.7, and 7.2 MPa, respectively. These results indicate that soaking weakens the resistance of the rock to fracture initiation and failure, demonstrating that soaking is an important factor contributing to the deterioration of the mechanical properties of the marker bed. Because all specimens used the same cement-wrapped configuration, the measured pressure changes mainly reflect the relative weakening trend under different soaking times.

To account for the influence of water induced weakening of the rock mass on casing failure, the changes in fracture initiation pressure under different soaking conditions were converted into equivalent strength parameters in the Drucker-Prager model. Fracture initiation pressure was used as the calibration target. The direct fracturing condition was taken as the reference state, and the equivalent strength parameters under different soaking histories were determined by proportionally reducing the baseline strength parameters according to the experimental decrease in fracture initiation pressure relative to the reference condition. This conversion is an engineering-equivalent calibration rather than a direct measurement of cohesion or friction angle. It assumes that, under identical specimen geometry and loading boundaries, the normalized decrease in fracture initiation pressure represents the overall weakening degree of the marker bed. Therefore, these equivalent parameters mainly describe macroscopic strength degradation and cannot fully capture tensile failure, microcrack evolution, or bedding-controlled anisotropic responses.The damage factor definition formula is


$$\begin{array}{c} D\left(t\right)=1-\frac{P\left(t\right)}{P_{\text{0}}} \end{array}$$ (1)

where D(t) is the damage factor at soaking time *t*, dimensionless; P(t) is the fracture initia*t*ion pressure at soaking time *t*, MPa; $P_{\text{0}}$ is the fracture initiation pressure under the direct fracturing condi*t*ion, MPa; and *t* is the soaking time. Accordingly, the equivalent cohesion of the marker bed rock at soaking time *t* can be expressed as:


$$\begin{array}{c} c\left(t\right)=c_{\text{0}}\left[1-D\left(t\right)\right]=c_{\text{0}}\frac{P\left(t\right)}{P_{\text{0}}} \end{array}$$ (2)

where $c_{\text{0}}$ is the baseline cohesion in the unsoaked state, MPa; c(t) is the equivalent cohesion obtained by inversion constrained by the hydraulic fracturing test after soaking time *t*, MPa. The dynamic shear strength criterion corrected by the damage factor is wri*tt*en as follows:


$$\begin{array}{c} \tau_{f}=c\left(t\right)+\sigma_{n}tan\varphi \left(t\right) \end{array}$$ (3)

where $\tau_{f}$ is the shear strength, MPa; φ(t) is the equivalent internal friction angle at soaking time *t*, rad; and $\sigma_{n}$ is the normal stress, MPa. The expression for the equivalent friction angle is given by:


$$\begin{array}{c} \varphi \left(t\right)=\varphi_{\text{0}}\left(1-D\left(t\right)\right) \end{array}$$ (4)

where $\varphi_{\text{0}}$ is the baseline internal friction angle in the unsoaked state, rad. Shear slip occurs along the bedding plane when the actual shear stress on the bedding plane satisfies $\tau >\tau_{f}$.

This laboratory boundary differs from the in-situ condition in stress anisotropy, formation heterogeneity, and long term seepage. Therefore, the laboratory fracture initiation pressure was used for equivalent weakening calibration rather than for directly defining field injection pressure limits. Specifically, it represents the local failure pressure of cement wrapped core specimens under controlled small scale loading conditions, whereas the field scale injection pressure limit represents an operational pressure threshold constrained by well scale pore pressure diffusion, formation deformation, and casing drift diameter reduction. These two pressures therefore have different physical meanings and should not be directly compared in magnitude.

## 3. Mechanical characterization

Casing failure under marker bed water invasion is essentially the result of the progressive evolution of the coupled formation and casing response. Water invasion increases pore pressure and alters the overall mechanical properties of the rock mass, thereby intensifying deformation nonuniformity in the formation near the marker bed. This process ultimately manifests in the wellbore casing as local stress concentration, plastic accumulation, and drift diameter loss. In view of this research scale and objective, mechanical characterization methods were established for both the rock mass and the casing. The former is used to describe effective stress evolution, overall strength degradation, and yielding behavior under marker bed water invasion, whereas the latter is used to characterize the yielding and failure response of the casing under nonuniform formation deformation.

### 3.1 Rock constitutive model and yield criterion

After water invasion of the marker bed, fluid infiltration increases pore pressure and alters the effective stress state of the rock mass. According to effective stress theory, the effective stress of the rock mass can be expressed as [27–29]:


$$\begin{array}{c} \sigma^{\prime }=\sigma -\alpha p𝐈 \end{array}$$ (5)

where $\sigma^{\prime }$ is the effective stress tensor, MPa; σ is the total stress tensor, MPa; *p* is the pore pressure, MPa; α is the Biot coefficient, dimensionless; and 𝐈 is the second order identity tensor, dimensionless.

At the continuum scale, the strength evolution of the marker bed after water invasion can be regarded as a transition from an elastic state to a plastically weakened state under triaxial stress conditions. Here, the Drucker–Prager model is used as a well-scale equivalent model rather than an explicit anisotropic bedding model. The equivalent cohesion and friction angle represent the averaged shear resistance of the weakened marker bed and its weak interfaces [28,30,31]. The cohesion degradation induced by water invasion is described by the time dependent equivalent cohesion correction relation established in Section 2.2. Accordingly, the yield function of the marker bed rock mass under water invasion conditions can be written as:


$$\begin{array}{c} F=\sqrt{J_{\text{2}}}+\frac{2sin\varphi \left(t\right)}{\sqrt{\text{3}}\left(3-sin\varphi \left(t\right)\right)}I_{\text{1}}-\frac{6c\left(t\right)cos\varphi \left(t\right)}{\sqrt{\text{3}}\left(3-sin\varphi \left(t\right)\right)}=0 \end{array}$$ (6)

where $J_{\text{2}}$ is the second invariant of the effective deviatoric stress, MPa^2^; and $I_{\text{1}}$ is the first invariant of the effective stress, MPa. To describe the evolution of plastic deformation after yielding, a non associated flow rule is adopted [18,21]. The corresponding plastic potential function is written as:


$$\begin{array}{c} G=\sqrt{J_{\text{2}}}+bI_{\text{1}} \end{array}$$ (7)

where b is a parameter related to the dilation angle, dimensionless.

### 3.2 Casing yielding and failure

The geomechanical response induced by marker bed water invasion is ultimately transferred to the wellbore casing through nonuniform formation deformation, causing local bending, stress concentration, and plastic accumulation. For the purpose of analyzing casing integrity at the well scale, the casing is treated as an elastoplastic steel body subjected to external formation loads [17–19]. Its stress strain relationship is expressed as:


$$\begin{array}{c} \sigma_{c}={𝐃}_{e}:\left(\varepsilon -\varepsilon^{p}\right) \end{array}$$ (8)

where $\sigma_{c}$ is the casing stress tensor, MPa; ${𝐃}_{e}$ is the elastic stiffness matrix, MPa; ε and $\varepsilon^{p}$ are the total strain tensor and plastic strain tensor, respectively, dimensionless. Since the casing material is ductile metal, the von Mises equivalent stress is adopted to characterize its yielding risk [30,31]:


$$\begin{array}{c} \sigma_{\text{vm}}=\sqrt{\frac{\text{3}}{\text{2}}𝐬:𝐬} \end{array}$$ (9)

where $\sigma_{\text{vm}}$ is the von Mises equivalent stress of the casing, MPa; and 𝐬 is the deviatoric stress tensor, MPa.

In view of the objectives of the numerical analysis, the maximum Mises stress, maximum equivalent plastic strain, and minimum drift diameter are selected as the three characterization indices for marker bed related casing failure. The maximum Mises stress is used to reflect the stress level at critical locations, the maximum equivalent plastic strain is used to characterize the accumulation of irreversible damage, and the minimum drift diameter is used to evaluate wellbore integrity and drift diameter retention capacity. The first two indices mainly describe the development of local casing damage, whereas the minimum drift diameter directly reflects wellbore operability and engineering serviceability. Based on the passability requirements of downhole tools used in the study block, a minimum drift diameter of 95 mm is adopted as the operability criterion for wellbore integrity evaluation. The following analysis of the safe pressure window and zonal pressure control is carried out on the basis of this drift diameter threshold.

## 4. Numerical model establishment and validation

### 4.1 Numerical model establishment

**Fig 4:**
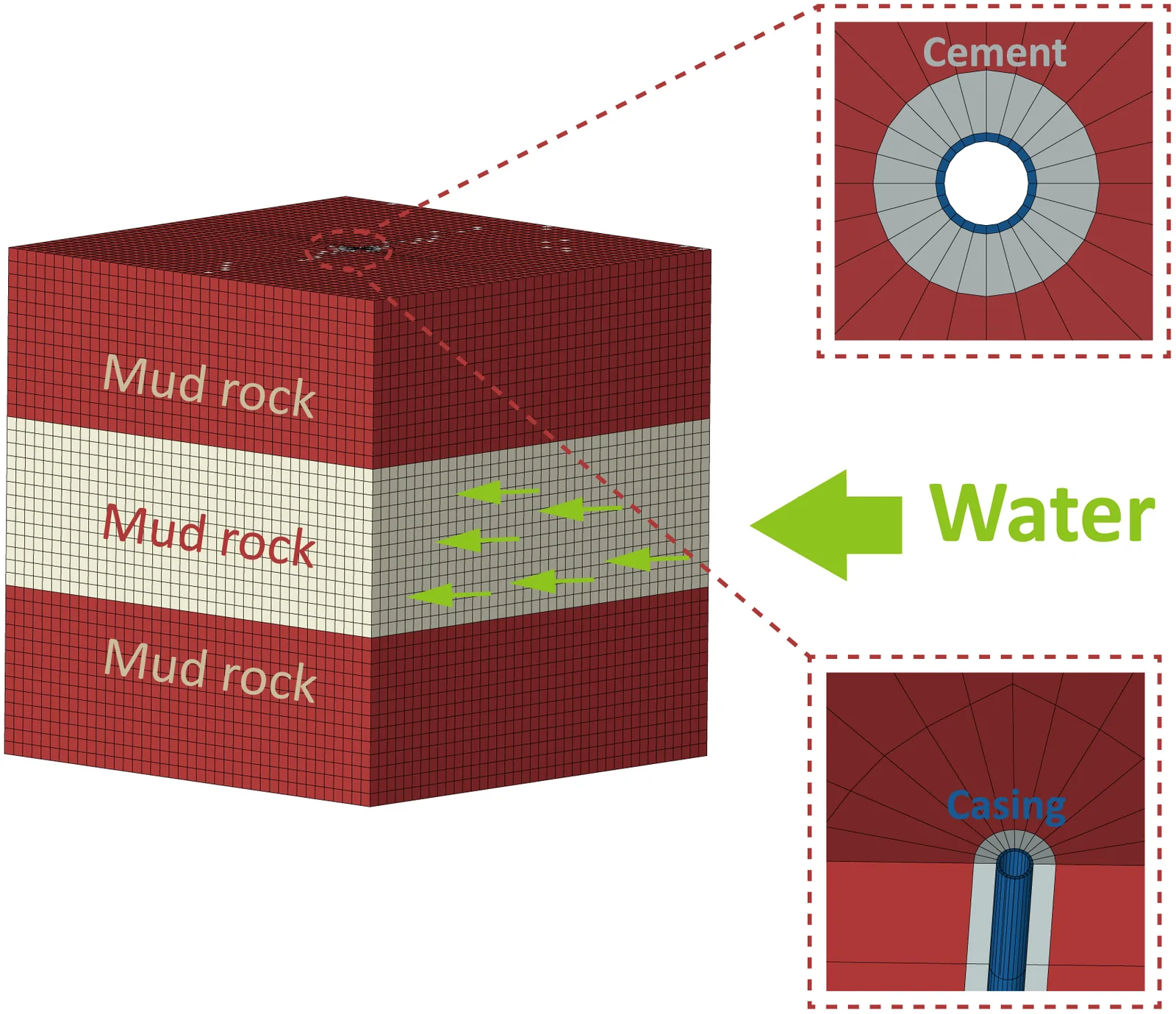
Schematic of the numerical model.

In this study, a simulation model was established for the marker bed developed in the mid-depth interval of the Sazhong development area of the Daqing Oilfield. Lithologic and logging data show that the formation in this area has a vertical sequence of mudstone, marker bed, and mudstone. The marker bed is mainly composed of oil shale and argillaceous shale and is about 10 m thick. It is confined above and below by mudstone with relatively strong plasticity. Because bedding planes and weak interfaces are well developed, the marker bed exhibits significant differences in stiffness and strength from the surrounding rock and therefore serves as the main controlling interval for casing shear failure [1,6,7,25,32]. Based on these stratigraphic characteristics, a three dimensional fluid-solid coupling numerical model of the wellbore, cement sheath, and formation was established in Abaqus [31,32] to simulate the stress and deformation of the casing under marker bed water invasion. In the present model, the casing, cement sheath, and formation were assumed to be continuously bonded, and interface debonding or micro-annuli were not explicitly simulated. This simplification was adopted to focus on the effect of marker bed weakening and nonuniform formation deformation on casing drift-diameter reduction.The model structure is shown in Fig 4. The marker bed is located in the middle of the model, with mudstone surrounding rock above and below, and the wellbore is positioned at the model center. This configuration can reasonably capture the coupled relationship among seepage, stress, and deformation near the marker bed.

To balance computational accuracy and efficiency, the computational domain was set to 30 m × 30 m × 30 m on the basis of preliminary model calculations. The marker bed thickness was set to 10 m, with 10 m mudstone layers above and below. The wellbore was positioned along the central axis of the model. The casing had an outer diameter of 139.7 mm and a wall thickness of 10 mm, and it was externally surrounded by a cement sheath with a thickness of 200 mm. The stratigraphic structure, wellbore dimensions, and boundary conditions of the model were determined according to the actual well conditions of Well 00-P2 and the development characteristics of the marker bed in which it is located. The in situ stress conditions were mainly defined on the basis of field log interpretation, whereas mechanical parameters such as rock density, elastic modulus, Poisson’s ratio, and shear modulus were determined from laboratory tests. The model with an injection pressure of 30 MPa, a marker-bed thickness of 10 m, a dip angle of 0°, and a permeability of 8.64 mD was defined as the base case. Mesh sensitivity was evaluated by comparing the baseline mesh, with a global seed size of 0.75 m and 63,804 elements, with a refined mesh, with a global seed size of 0.60 m and 129,336 elements. Only the mesh density was changed, while all other model settings remained unchanged. The relative differences in minimum drift diameter, maximum Mises stress, and maximum equivalent plastic strain were 2.47%, 1.36%, and 2.93%, respectively. Since all differences were within 3%, the baseline mesh was considered sufficient and was used in the subsequent simulations.The relevant parameters are summarized in Table 1.

**Table 1:** Geological parameters of a block in the Sazhong development area of the Daqing Oilfield.

Well depth (m)	Minimum horizontal principal stress (MPa)	Maximum horizontal principal stress (MPa)	Vertical stress (MPa)	Rock density(g·cm^-3^)	Poisson’s ratio	Elastic modulus (GPa)	Shear modulus (GPa)
859.5	15.4	19.4	15.4	2.1	0.35	7.4	2.7

### 4.2 Field data validation

**Fig 5:**
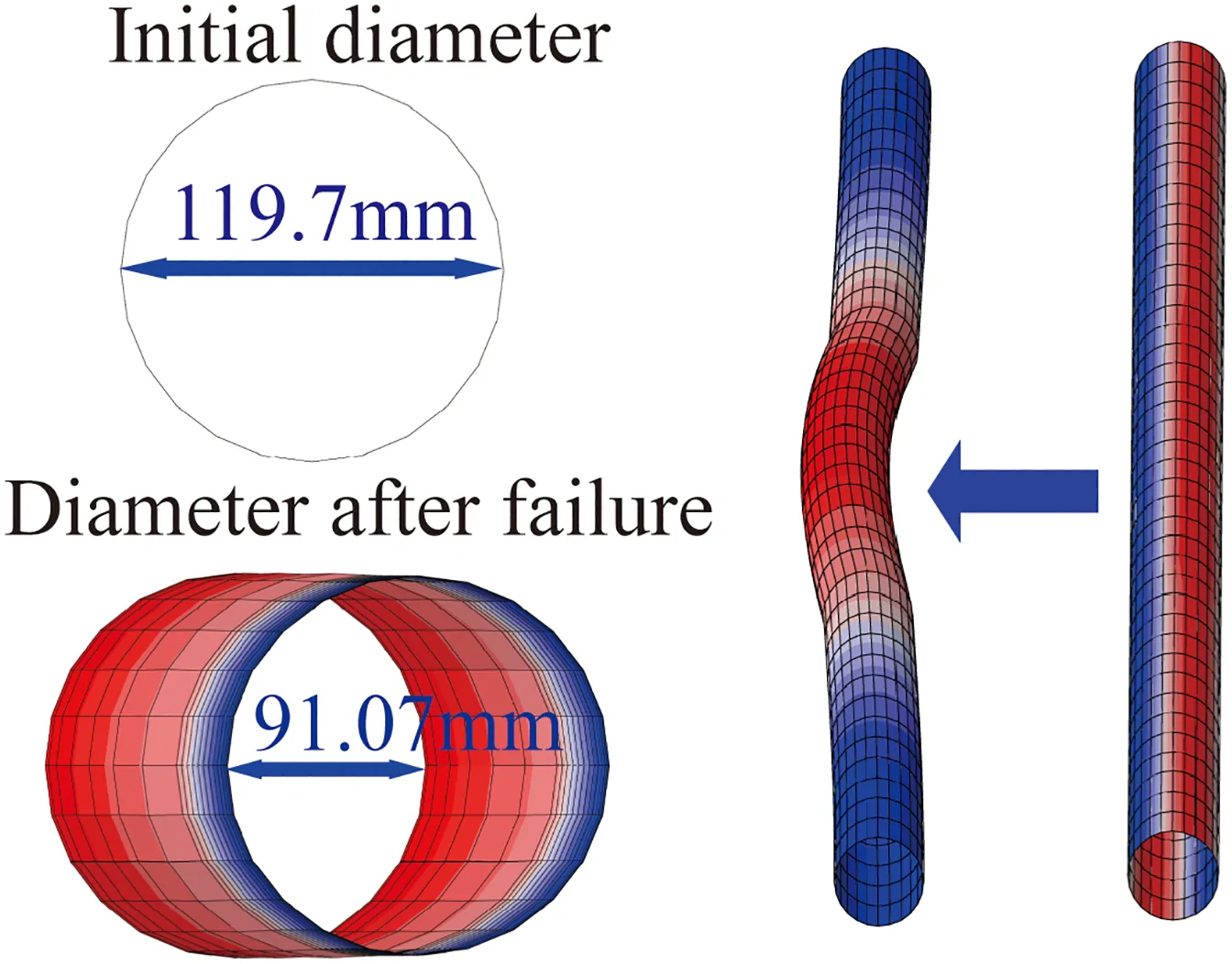
Contour plot of simulated casing deformation.

The test data from casing failure Well 00-P2 in the Sazhong development area of the Daqing Oilfield were selected for model validation. The original casing drift diameter of this well was 119.7 mm, and the monitored drift diameter decreased to 93.2 mm after casing failure. The calculated horizontal casing deformation was 26.7 mm, and the deformation was mainly concentrated within the marker bed interval. A corresponding numerical model was established on the basis of field logging data and laboratory test parameters, and the calculation was performed using a measured water pressure of 30 MPa at the casing deformation interval. The simulation results are shown in Fig 5. Within the marker bed interval, the casing inner diameter decreased from 119.7 mm to 91.07 mm, and the simulated horizontal casing deformation was 28.63 mm. The relative error was 7.2%, indicating that the model reasonably reproduces the observed casing deformation at the failure interval. It should be noted that this validation is based on one representative casing-failure well. Therefore, the model is mainly applicable to wells with similar marker-bed lithology, stratigraphic configuration, casing structure, and water-invasion conditions, and further validation using additional field cases is still needed before broader application to other blocks or geological settings.

## 5. Results and discussion

### 5.1 Analysis of casing failure mechanism

**Fig 6:**
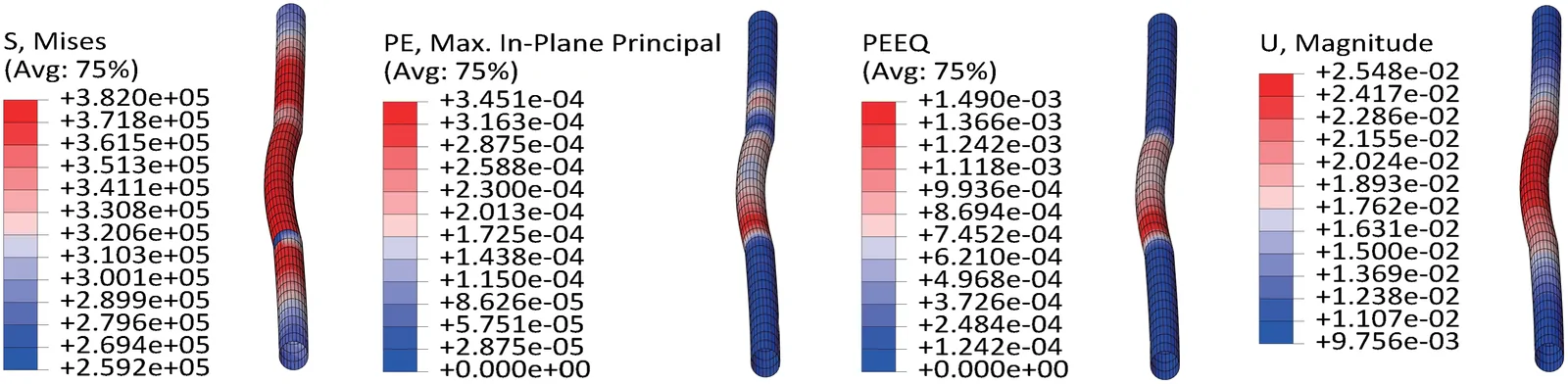
Contour plots of casing failure evolution under the base case. **(a)** Mises stress; **(b)** plastic strain; **(c)** accumulated plastic strain; and **(d)** displacement.

To analyze the spatial distribution and temporal evolution of the casing failure response under marker bed water invasion, the validated numerical simulation results for Well 00-P2 were used to extract the stress, plastic strain, and displacement distributions after casing deformation, as shown in Fig 6. The results show that the casing undergoes significant bending deformation after marker bed water invasion. As shown in Fig 6(a), the maximum Mises stress is concentrated in the bent section of the casing and is mainly located at the interfaces between the marker bed and the overlying and underlying surrounding rocks. As shown in Fig 6(b) and 6(c), plastic deformation also first appears near these interfaces, and the maximum plastic accumulation occurs at the lower interface.

**Fig 7:**
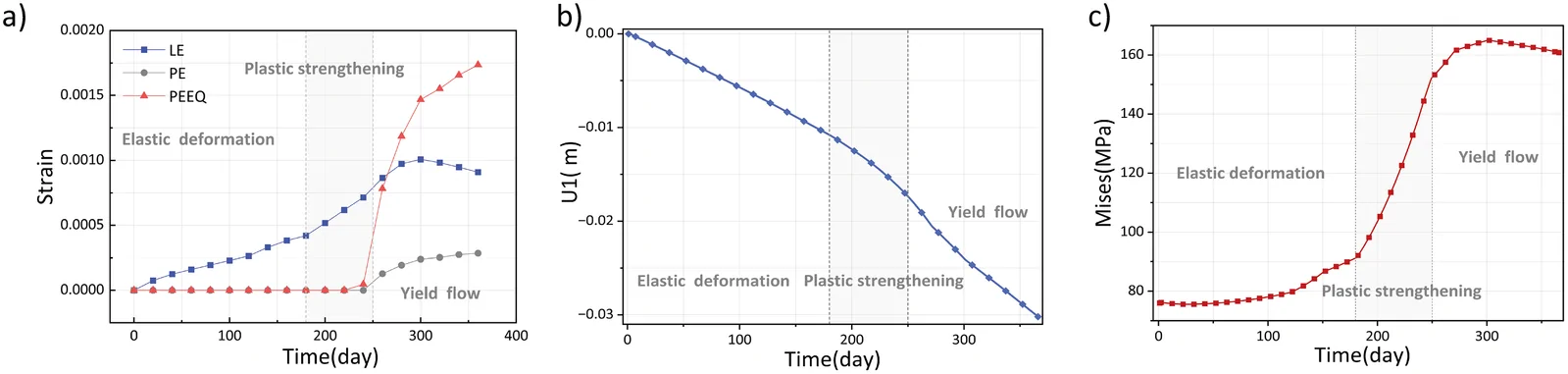
Time history responses at the key point under the base case. **(a)** elastic and plastic strain; **(b)** displacement; and **(c)** Mises stress.

The time histories of elastic strain, plastic strain, displacement, and Mises stress at the point of equivalent plastic strain were extracted, and the results are shown in Fig 7. The results show that casing failure undergoes a distinct three stage evolution process. In the first stage, the casing mainly exhibits elastic deformation. Elastic strain gradually increases, whereas plastic strain remains essentially zero. In the second stage, as pore-pressure diffusion and formation loading continue to develop, the external load on the casing keeps increasing. After about 250 d, the casing reaches its yield point and enters a stage of accelerated plastic development, during which the Mises stress and equivalent plastic strain rise rapidly. In the later stage, the local plastic zone continues to expand, and the casing enters a yielding flow stage. The stress level tends to stabilize, whereas displacement and plastic accumulation continue to develop, ultimately resulting in pronounced drift diameter reduction. In this simulation, the laboratory-derived damage factor was assigned as an equivalent weakened state, while the time evolution in Fig 7 mainly reflects the coupled pore-pressure diffusion and casing deformation response.

Casing deformation therefore follows a three stage evolution process, including elastic deformation, accelerated plastic development, and yielding flow. Pore pressure increase and water induced strength degradation play different but coupled roles in this process. The increase in pore pressure reduces the effective normal stress and weakens the mechanical constraint at the marker bed mudstone interfaces, thereby promoting interfacial slip and nonuniform deformation. In contrast, water-induced strength degradation reduces the equivalent cohesion, frictional resistance, and supporting stiffness of the marker bed, making the weakened interval more prone to plastic deformation. The combined effect of these two processes increases the deformation contrast between the marker bed and the surrounding mudstone, localizes the external load transferred to the casing, and eventually leads to casing bending, plastic accumulation, and drift diameter reduction.

### 5.2 Single factor response analysis

**Fig 8:**
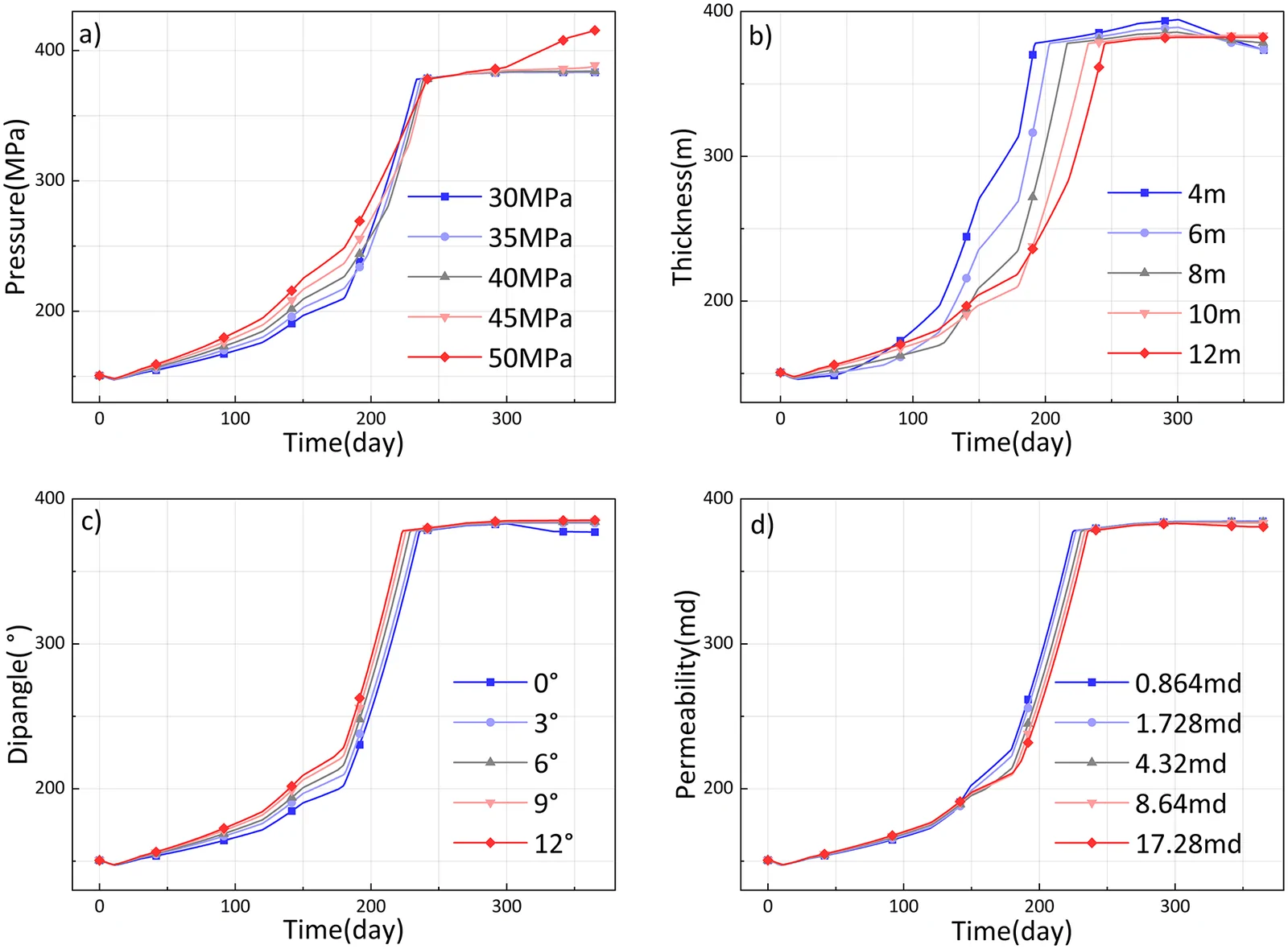
Response of maximum Mises stress to four influencing factors. **(a)** injection pressure; **(b)** marker bed thickness; **(c)** marker bed dip angle; and **(d)** marker bed permeability.

Based on the base case, single factor simulations were further conducted for four variables, including injection pressure, marker bed thickness, marker bed dip angle, and marker bed permeability. The maximum Mises stress, maximum equivalent plastic strain, and minimum drift diameter were taken as the response indices. Since the first two indices can reflect the initiation and accumulation of casing failure, the time histories of maximum Mises stress and maximum equivalent plastic strain under different factors were first compared. The results are shown in Fig 8.

Fig 8(a)–8(d) show the response of maximum Mises stress to injection pressure, marker bed thickness, marker bed dip angle, and marker bed permeability, respectively. As shown in Fig 8(a), the maximum Mises stress increases with increasing injection pressure. When the injection pressure rises from 45 MPa to 50 MPa, the increase becomes particularly pronounced, indicating that higher injection pressure markedly amplifies stress concentration at critical locations. As shown in Fig 8(b), with increasing marker bed thickness, the onset of rapid stress growth is progressively delayed, and the maximum Mises stress gradually decreases. This indicates that a thinner marker bed is more likely to cause stress concentration between the upper and lower interfaces. As shown in Fig 8(c), the maximum Mises stress generally increases with increasing marker bed dip angle. When the dip angle increases from 0° to 3°, the maximum Mises stress rises sharply. When the dip angle further increases from 3° to 12°, the increase becomes much less pronounced. This suggests that, compared with a horizontal marker bed, initial tilting significantly enhances stress concentration within the marker bed, whereas further increases in dip angle have a relatively limited effect. As shown in Fig 8(d), the maximum Mises stress gradually decreases with increasing marker bed permeability, but the overall variation remains small, indicating that marker bed permeability has only a minor influence on stress concentration.

**Fig 9:**
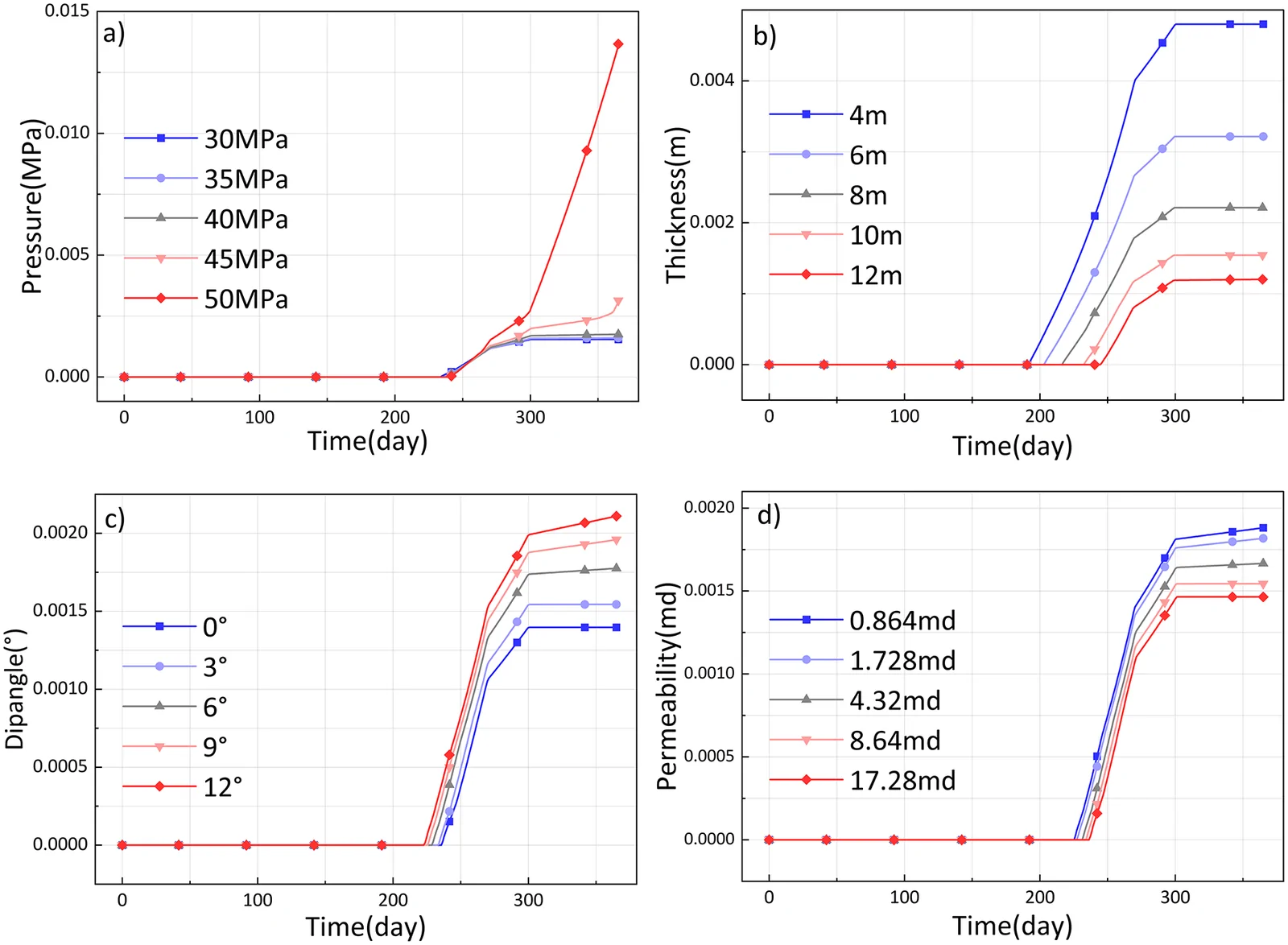
Response of maximum equivalent plastic strain to four influencing factors. **(a)** injection pressure; **(b)** marker bed thickness; **(c)** marker bed dip angle; and **(d)** marker bed permeability.

Fig 9(a)–9(d) show the response of maximum equivalent plastic strain to injection pressure, marker bed thickness, marker bed dip angle, and marker bed permeability, respectively. As shown in Fig 9(a), the onset time of plastic deformation is relatively similar under different injection pressures, whereas the maximum equivalent plastic strain increases continuously with increasing injection pressure. When the injection pressure rises from 45 MPa to 50 MPa, the maximum equivalent plastic strain increases markedly. This trend is consistent with that observed for the maximum Mises stress. As shown in Fig 9(b), with increasing marker bed thickness, the onset of plastic deformation is significantly delayed, and the maximum equivalent plastic strain gradually decreases. These results indicate that marker-bed thickness affects local damage indices and global operability differently: increasing thickness alleviates local interfacial stress concentration to some extent, but reduces the overall constraint stiffness of the weakened interval, resulting in a larger drift-diameter loss. As shown in Fig 9(c), with increasing marker bed dip angle, the onset of plastic deformation occurs progressively earlier, and the maximum equivalent plastic strain gradually increases. As shown in Fig 9(d), with increasing marker bed permeability, the onset of plastic deformation is progressively delayed, and the maximum equivalent plastic strain gradually decreases.

**Fig 10:**
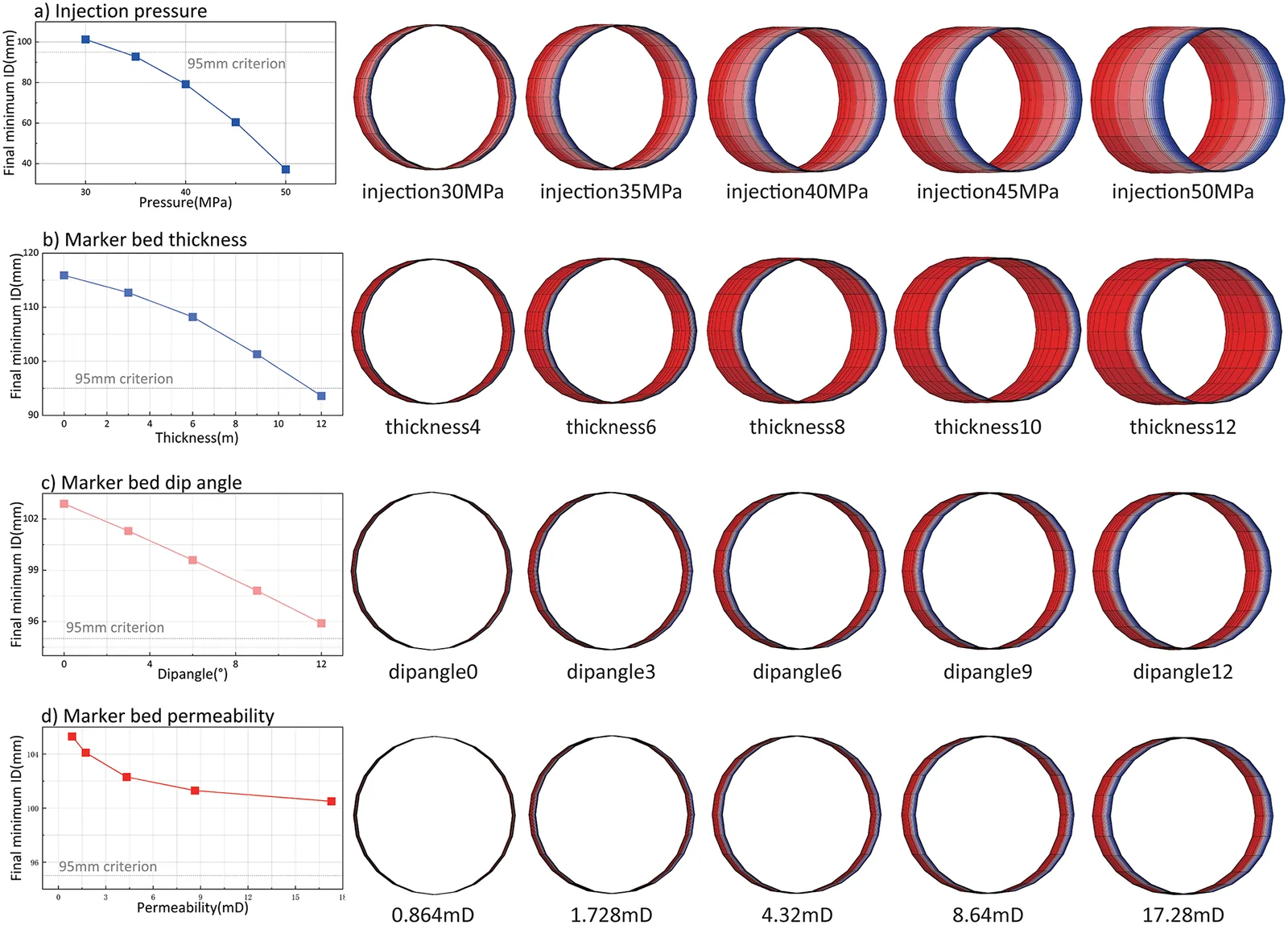
Effects of four factors on the minimum drift diameter.

The minimum drift diameter is a direct engineering index for evaluating wellbore integrity and operability. Fig 10 shows the response of minimum drift diameter to injection pressure, marker bed thickness, marker bed dip angle, and marker bed permeability. The results show that the minimum drift diameter decreases rapidly with increasing pressure and crosses the 95 mm wellbore operability threshold between 30 and 35 MPa. This indicates that increasing injection pressure significantly narrows the allowable pressure operating window. The minimum drift diameter continues to decrease as marker bed thickness increases. Although a thicker marker bed produces less accumulated plastic deformation at the interfaces, increasing marker bed thickness reduces its overall stiffness. As a result, its ability to restrain radial casing deformation weakens, the lateral displacement increases, and the minimum drift diameter continues to decrease. As the marker bed dip angle increases, the minimum drift diameter decreases in an approximately linear manner and is reduced to 95.9 mm when the dip angle reaches 12°. As marker bed permeability increases, the minimum drift diameter gradually decreases, but the rate of reduction progressively diminishes. The overall decrease remains limited, and the predicted value still stays above the 95 mm wellbore operability threshold.

In summary, the single factor response analysis shows that all four factors affect casing failure. Among them, injection pressure has the strongest influence and exhibits a pronounced nonlinear effect. Marker bed thickness exerts a dual influence on both casing failure and drift diameter, whereas marker bed dip angle and permeability have relatively smaller effects.

### 5.3 Sensitivity analysis of factors affecting casing failure

To consistently compare the influence of different controlling factors on the casing failure response, a comprehensive evaluation method combining dimensionless sensitivity coefficients and the entropy weight method is adopted on the basis of the single factor simulation results [33–35]. The controlling factors considered are injection pressure, marker bed thickness, marker bed dip angle, and marker bed permeability, whereas the response indices are maximum Mises stress, maximum equivalent plastic strain, and minimum drift diameter.

The dimensionless sensitivity coefficient of the *i*th controlling factor with respect to the *j*th response index is defined as:


$$\begin{array}{c} S_{ij}=\frac{(y_{ij,max}-y_{ij,min})/\overset{―}{y_{ij}}}{(x_{i,max}-x_{i,min})/\overset{―}{x_{i}}} \end{array}$$ (10)

where $S_{ij}$ is the dimensionless sensitivity coefficient; $x_{i,max}$, $x_{i,min}$, and $\overset{―}{x_{i}}$ are the maximum, minimum, and average values of the controlling factor in the single factor simulations, respectively; $y_{ij,max}$, $y_{ij,min}$, and $\overset{―}{y_{ij}}$ are the maximum, minimum, and average values of the corresponding response index, respectively.

On this basis, to comprehensively account for the importance of the three response indices in evaluating casing failure, the entropy weight method is adopted to determine the weight of each response index, and the comprehensive sensitivity index of the *i*th controlling factor is defined as:


$$\begin{array}{c} C_{i}=\sum_{j=1}^{n}\omega_{j}S_{ij} \end{array}$$ (11)

where $C_{i}$ is the comprehensive sensitivity index; and $\omega_{j}$ is the weight of the *j*th response index, determined by the entropy weight method.

**Fig 11:**
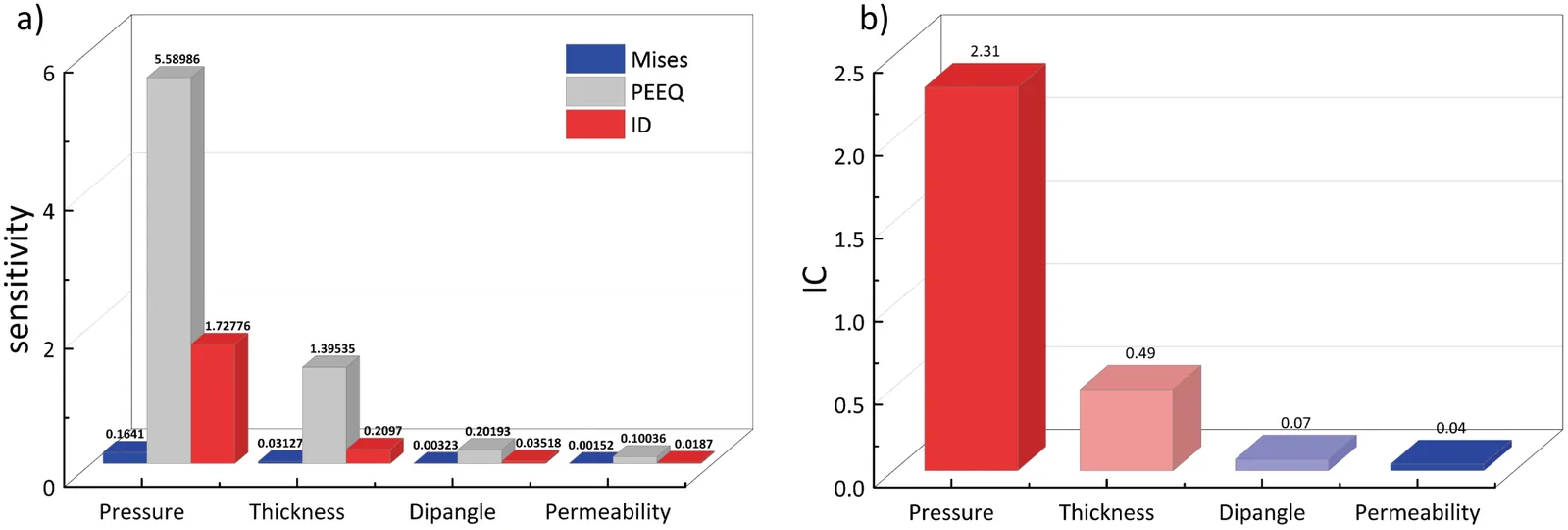
Sensitivity analysis results. **(a)** dimensionless sensitivity coefficients of four main controlling factors for maximum Mises stress, maximum equivalent plastic strain, and minimum drift diameter; and **(b)** comprehensive sensitivity index.

Fig 11(a) shows the influence of the four factors on the three response indices, namely maximum Mises stress, maximum equivalent plastic strain, and minimum drift diameter. Among them, injection pressure exerts the strongest influence on all three indices and has the greatest effect on accumulated plastic strain. Marker bed thickness also shows a strong influence on accumulated plastic strain, whereas marker bed dip angle and marker bed permeability have relatively smaller effects on all three response indices.

Fig 11(b) shows the comprehensive sensitivity indices of the four factors. The influence of these factors on casing failure follows the order of injection pressure > marker bed thickness > marker bed dip angle > marker bed permeability, with corresponding sensitivity indices of 2.31, 0.49, 0.07, and 0.04, respectively. The sensitivity index of injection pressure is far greater than those of the other three factors, indicating that injection pressure is the dominant controlling factor for casing failure.

## 6. Recommendations for casing failure prevention and control

### 6.1 Risk identification and pressure limit criterion

According to the sensitivity analysis results presented in Section 5.3, marker-bed-related casing failure is jointly controlled by injection pressure, marker bed thickness, dip angle, and permeability. Because injection pressure is the most sensitive and directly adjustable factor, the sensitivity analysis provides the basis for converting the casing deformation response into an operational pressure-limit criterion. Therefore, the engineering identification of marker bed related casing failure should not rely on single factor judgment alone. Instead, a pressure limit criterion should be established with wellbore integrity indices as the core. In this section, a minimum drift diameter of no less than 95 mm is adopted as the engineering control threshold for wellbore operability. According to their relative influence, injection pressure is treated as the direct control variable, whereas marker bed thickness, dip angle, and permeability are treated as geological constraint parameters. This threshold is treated as a deterministic operability requirement based on downhole tool passability, while measurement uncertainty and additional safety factors are not explicitly modeled in the present analysis.

Let the marker bed condition x be characterized by thickness, dip angle, and permeability. Then, the upper limit of injection pressure under this condition is defined as:


$$\begin{array}{c} P_{max}\left(x\right)=max\left\{P|\Phi_{min}\left(P,x\right)\geq 95\text{mm}\right\} \end{array}$$ (12)

where $\Phi_{min}\left(P,x\right)$ is the response value of the minimum drift diameter with respect to injection pressure *P* under condition x, obtained by piecewise linear interpolation from the numerical results of discrete pressure cases. If a given condition does not satisfy $\Phi_{min}\geq 95\text{mm}$ under the reference pressure, its upper pressure limit should be lower than that reference pressure. Therefore, the minimum drift diameter constraint can be directly converted into an operational pressure window.

### 6.2 Zonal pressure control results and engineering recommendations

**Fig 12:**
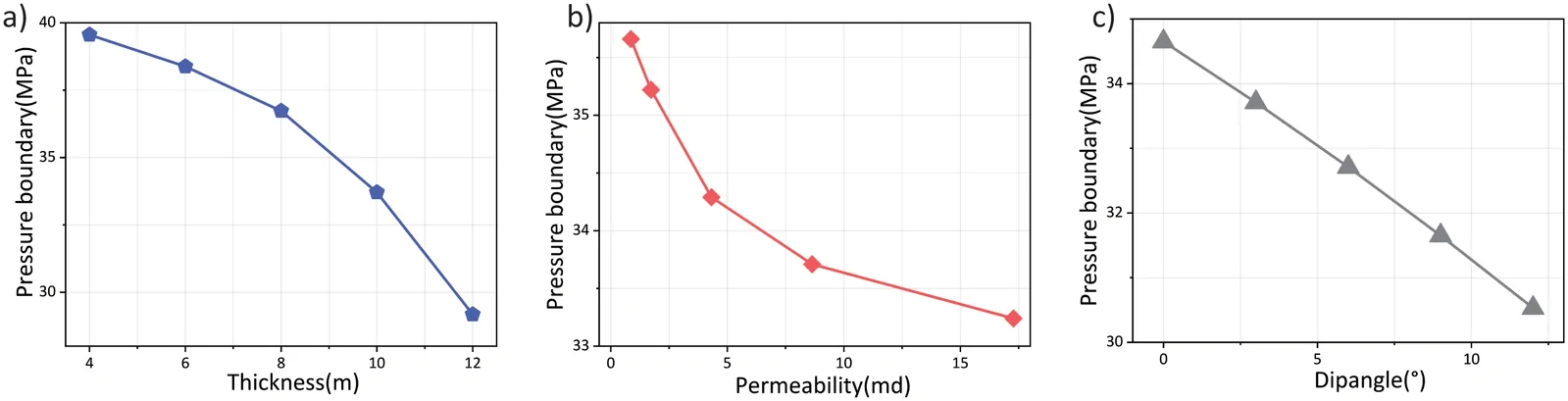
Upper injection pressure limits under different marker bed conditions. **(a)** upper injection pressure limits for different marker bed thicknesses; **(b)** upper injection pressure limits for different marker bed dip angles; and **(c)** upper injection pressure limits for different marker bed permeabilities.

The upper pressure limits obtained from Eq. (12) are shown in Fig 12. Overall, the allowable upper limit of injection pressure decreases with increasing marker bed thickness, dip angle, and permeability, although the influence of these three factors differs significantly. When the marker bed thickness increases from 4 m to 12 m, the upper injection pressure limit decreases from about 39.6 MPa to 29.2 MPa. When the dip angle increases from 0° to 12°, the upper pressure limit decreases from about 34.6 MPa to 30.5 MPa. When permeability increases from 0.864 mD to 17.28 mD, the upper pressure limit decreases from about 35.7 MPa to 33.2 MPa. These results indicate that marker bed thickness has the strongest controlling effect on the injection pressure limit, followed by dip angle, whereas permeability has the weakest effect.

For practical field application, the marker bed was divided into nine zones, denoted as Z1 to Z9, and the upper injection pressure ranges corresponding to three permeability levels were determined for each zone. The results are listed in Table 2. Overall, the thin marker bed zones (Z1 to Z3) have upper pressure limits of 35–40 MPa under different dip angle and permeability conditions, indicating that this type of marker bed has a relatively large drift diameter margin. The pressure window of the medium thickness zones (Z4 to Z6) becomes markedly narrower, and under most conditions the upper pressure limit decreases to 30–35 MPa. Only when the dip angle is small and the permeability is low can it still remain within 35–40 MPa. The thick marker bed zones (Z7 to Z9) shift further into the low pressure range, and the upper pressure limit under most conditions falls below 30 MPa. These zones should therefore be regarded as key risk areas.

**Table 2:** Marker bed zoning and upper injection pressure limits.

Zone	Thickness (m)	Dip angle (°)	Low Permeability	Medium Permeability	High Permeability
**Z1**	8	3	[35,40)	[35,40)	[35,40)
**Z2**	8	6	[35,40)	[35,40)	[35,40)
**Z3**	8	9	[35,40)	[35,40)	[35,40)
**Z4**	10	3	[35,40)	[30,35)	[30,35)
**Z5**	10	6	[35,40)	[30,35)	[30,35)
**Z6**	10	9	[30,35)	[30,35)	[30,35)
**Z7**	12	3	[30,35)	<30	<30
**Z8**	12	6	[30,35)	<30	<30
**Z9**	12	9	<30	<30	<30

Therefore, for the thin marker bed zones, 35 MPa can be adopted as the routine injection pressure control value, with particular emphasis on suppressing short term pressure fluctuations. For the medium thickness zones, the injection pressure should preferably be controlled within 30–35 MPa. Stable operation should be achieved primarily through injection allocation and zonal regulation, rather than by simply increasing injection pressure. For the thick marker bed zones, reduced pressure operation should be adopted as the primary strategy, with the injection pressure controlled below 30 MPa. When necessary, packer based isolation and related measures should be applied to reduce the adverse effect of local pressure concentration on casing integrity. Overall, the engineering control of marker bed related casing failure should not rely on a unified upper pressure limit. Instead, zonal pressure control should be established according to marker bed thickness and dip angle and then adjusted according to permeability, so as to achieve differentiated pressure control and wellbore integrity management.

## 7. Conclusions

This study investigated wellbore casing failure induced by marker bed water invasion during the later stage of compound flooding development in oilfields. By combining field monitoring, cement wrapped hydraulic fracturing tests on marker bed cores, mechanical characterization of water induced weakening, and fluid-solid coupling numerical simulation, the response characteristics, controlling factors, and engineering control methods of casing failure were systematically analyzed. The main conclusions are as follows:

(1) Field monitoring shows that anomalous water invasion in the marker bed corresponds clearly to the intervals of casing failure. Distributed fiber optic temperature monitoring in two casing failure wells indicates that water invasion anomalies near the marker bed intensify continuously with time. In the representative well, the anomalous water invasion interval near 859.5 m is highly consistent with the casing failure interval later identified by logging, indicating that marker bed water invasion is an important field manifestation associated with casing failure.
(2) Laboratory soaking and refracturing tests show that the fracture initiation pressure of the marker bed rock decreases continuously with increasing soaking time, indicating that soaking significantly reduces the fracture initiation resistance and overall mechanical strength of the marker bed rock. The time dependent equivalent cohesion correction relation established on this basis can be used to characterize the water weakening process of the marker bed and to provide parameters for subsequent continuum scale yield analysis of the rock mass.
(3) After marker bed water invasion, casing failure is controlled by the coupling of pore-pressure increase and water-induced strength degradation, which promotes interfacial deformation incompatibility, casing bending, plastic accumulation, and drift-diameter reduction. For the representative well, the error between the calculated reduction in casing inner diameter and the field measurement is 7.2%, indicating that the proposed model has reasonable engineering applicability for this case. However, because the quantitative validation was conducted using a single representative well, further validation with additional field cases is required to improve its general applicability.
(4) The results of the single factor analysis and the comprehensive sensitivity ranking consistently indicate that the controlling factors follow the order of injection pressure > marker bed thickness > marker bed dip angle > marker bed permeability. Among them, injection pressure is the most direct control variable and determines the severity of failure, whereas marker bed thickness is the main structural factor; although increasing thickness tends to reduce local interfacial stress concentration, it leads to a greater loss of drift diameter and thus a more severe reduction in wellbore operability.
(5) With a minimum drift diameter of no less than 95 mm as the engineering criterion, an engineering method was established to calculate the upper injection pressure limit on the basis of discrete numerical results, and zonal pressure control recommendations were proposed. The results show that thin marker beds have a relatively large pressure margin, whereas the safe pressure window becomes markedly narrower for thick marker beds with high dip angles. Accordingly, it is recommended that field operations adopt a control strategy combining zonal pressure control with supporting operational measures so as to improve the specificity and operability of wellbore integrity management.
